# Final Femoral Rasp Predicts Intraoperative Stem Alignment in Primary Total Hip Arthroplasty Using a Cementless Triple-Tapered Stem

**DOI:** 10.1016/j.artd.2026.102186

**Published:** 2026-10-15

**Authors:** Yannick Alexander Ahlheit, Gautier Beckers, Dominic Simon, Salome Hagelstein, Mattia De Benedictis, Boris Michael Holzapfel, Lennart Marvin Schroeder

**Affiliations:** Musculoskeletal University Center Munich (MUM), Department of Orthopaedics, Trauma Surgery and Rehabilitative Medicine, LMU University Hospital Munich, Munich, Germany

**Keywords:** Total hip arthroplasty, Direct anterior approach, Alignment, Fluoroscopy, Rasp, Cementless

## Abstract

**Background:**

The final femoral rasp (FFR) serves as an intraoperative surrogate for positioning the final femoral implant (FFI) in total hip arthroplasty (THA), yet quantitative data on rasp–implant concordance for modern triple-tapered stems are limited. This study quantified positional differences between the final rasp and implant in primary cementless THA via the direct anterior approach (DAA).

**Methods:**

This prospective study included 63 THA procedures using the DAA. Intraoperative fluoroscopic images of FFR and FFI were analyzed in coronal and axial projections, measuring from lateral shoulder to the greater trochanter tip and from osteotomy plane to taper apex in coronal view, as well as angular deviation between rasp/implant axis and proximal femoral shaft axis in both views. Positional differences (Δ = FFR − FFI) were assessed.

**Results:**

The implant was positioned more proximally than the rasp (Δ greater trochanter–shoulder = 0.64 ± 2.51 mm, *P* = .048) and demonstrated valgus alignment relative to the rasp (Δ coronal angle = 0.63° ± 1.38°, *P* < .001), whereas no differences were observed for the remaining parameters.

**Conclusions:**

This intraoperative analysis demonstrated close positional agreement between FFR and FFI in a cementless, triple-tapered stem THA design implanted via the DAA. The FFI was positioned in valgus and at a more proximal level than the FFR. These differences were limited and likely attributable to minor intraoperative adjustment and design-related geometric differences. Overall, the findings support the reliability of the FFR as an intraoperative surrogate for implant positioning in cementless THA.

## Introduction

Accurate positioning of cementless femoral components in total hip arthroplasty (THA) is crucial for primary stability and ensuring long-term fixation [1]. Malposition may alter load transfer and increase micromotion, contributing to leg-length discrepancy, postoperative thigh pain, and aseptic loosening [[2], [3], [4]].

To prevent these complications, precise femoral broaching is essential, as it shapes the femoral canal, while the final rasp serves as an intraoperative surrogate for definitive implant positioning [[5], [6], [7]]. Accordingly, the position of the final trial rasp is intended to be adopted by the definitive femoral stem [7] to achieve correct alignment, depth, and rotational orientation.

However, quantitative data on potential rasp–implant mismatch remains limited [7,8] and no intraoperative studies have evaluated rasp–implant concordance in cementless triple-tapered stems, despite the popularity of contemporary wedge-taper stem designs [9].

Therefore, the present study evaluated the intraoperative relationship between the final rasp and implant positioning with positional differences quantified in the coronal and axial (Lauenstein) views.

We hypothesized that the final femoral rasp (FFR) and the final femoral implant (FFI) would not differ systematically in their final position.

## Material and methods

### Study design

This study was designed as an intrapatient–controlled radiographic analysis to assess the positional relationship between rasp and implant during primary cementless THA via the direct anterior approach (DAA). The study was approved by the local ethics committee (Ref. 24-0188) and conducted in accordance with the Declaration of Helsinki.

### Patient selection and demographics

All consecutive patients undergoing THA via the DAA with the AESCULAP® Primary CoreHip® System (B. Braun, Tuttlingen, Germany) between December 2023 and September 2025 were included in a prospectively maintained institutional arthroplasty database. Eligible cases required complete paired intraoperative fluoroscopic image series of rasp and implant in both planes meeting predefined quality criteria (Section 2.4). Furthermore, patients aged <18 years or with prior hip infection, previous surgery on the index joint, systemic neuromuscular disease, or pregnancy were excluded.

Of 245 procedures initially included in the arthroplasty database, 114 were excluded due to incomplete imaging and 69 for not meeting predefined quality criteria required to ensure measurement reliability.

Recruitment continued until the predefined power target was reached, resulting in 63 procedures.

Table 1 summarizes the baseline demographic characteristics of the patients.

**Table 1:** Patient demographics and baseline characteristics.

Variable	Value
Age, y	66.0 (IQR 54.5-76.5)
Sex	Men: 27 (42.9%), Women: 36 (57.1%)
Height, m	1.7 ± 0.1 (1.4-1.9)
Weight, kg	76.8 ± 16.7 (32.0-125.0)
BMI, kg/m^2^	26.1 (IQR 23.5-29.9)
ASA classification	I: 2 (3.2%), II: 25 (39.7%), III: 36 (57.1%)
Dorr classification	A: 34 (54%), B: 25 (39.7%), C: 4 (6.3%)
CTI	0.6 (IQR 0.5-0.6)
Laterality	Right: 36 (57.1%), Left: 27 (42.9%)

Data are presented as mean ± SD with ranges or median and interquartile range (25th–75th percentiles) for non-normally distributed variables.

BMI, body mass index; ASA, American Society of Anesthesiologists; SD, standard deviation; IQR, interquartile range.

Regarding procedural characteristics, 43 hips (68.3%) received a standard (132°) and 20 hips (31.7%) a varus (122°) neck variant. Stem sizes ranged from 0 to 9, with sizes 5 (22.2%), 6 (17.5%), and 7 (15.9%) being the most frequently used.

### Surgical technique

All procedures were performed in supine position via the DAA without a traction table. Following implantation of the acetabular component, the femoral preparation was performed manually using the manufacturer's sequential broaching system without pneumatic assistance. Broaching was continued until simultaneous axial and rotational stability of the final rasp was achieved according to the standardized institutional implantation protocol. The component stem used is an uncollared metaphyseal-loading implant; therefore, definitive seating is determined by metaphyseal press-fit rather than calcar contact. Preoperative digital templating was routinely performed to determine the planned femoral neck osteotomy, anticipated stem size, and expected implantation depth. The final rasp was retained for trial reduction and fluoroscopic assessment, followed by implantation of the definitive cementless stem and ceramic head with repeat fluoroscopy. Standard (132°) or varus (122°) neck variants were selected according to patients’ anatomy.

Six fellowship-trained arthroplasty surgeons performed the included procedures. Three surgeons, each with at least 5 years of independent arthroplasty practice following fellowship training, were classified as highly experienced, whereas three surgeons with fewer than 5 years of independent practice were classified as experienced. All surgeons routinely perform the direct anterior approach and followed an identical institutional surgical protocol throughout the study period.

### Imaging protocol and measurements

Intraoperative fluoroscopic images were obtained as part of standard clinical care using a standardized institutional imaging protocol and a mobile C-arm guided by the operating surgeon. Coronal images were acquired with approximately 15° of internal rotation of the lower extremity, whereas axial (Lauenstein) images were obtained with approximately 45° of hip flexion and abduction, first with the final rasp in situ and subsequently after definitive stem implantation. Fluoroscopy was performed with the highest degree of accuracy reasonably achievable under routine surgical conditions, using standardized limb positioning, implant-specific radiographic landmarks, and predefined projection-quality criteria. Only images fulfilling these criteria were included. Coronal projections required visualization of the rasp reference openings together with a horizontal implant shoulder, whereas axial projections required perpendicular visualization of the sagittal implant surface. Image comparability was additionally confirmed using implant-specific templates within mediCAD® (Version 7.0, Hectec GmbH, Altdorf, Germany), thereby minimizing rotational variability between the final rasp and definitive implant images. All radiographic measurements for the primary analysis were performed by 1 member of the orthopaedic research team experienced in radiographic assessment using mediCAD® software. Two additional investigators participated in the reliability analysis. No clinical radiologists were involved. Prior to study initiation, all observers underwent standardized training using a predefined measurement protocol.

Proximal femoral morphology was characterized using the cortical thickness index (CTI), measured 10 cm distal to the lesser trochanter as the ratio of cortical to total femoral diameter and classified according to Dorr, Faugere et al [10].

For both the FFR and the FFI, the longitudinal axis was defined using the corresponding mediCAD® template, according to the manufacturer’s implant geometry. In the coronal projection, this axis extended from the distal tip toward the lateral aspect of the proximal shoulder. The femoral shaft axis (FSA) was determined by connecting the midpoint of the intramedullary canal just below the lesser trochanter and the midpoint of the intramedullary canal at the mid-shaft level, as previously described by Yazdi, Nazarian et al [11].

As illustrated in Figure 1, the angular and linear parameters were measured to characterize the component positioning for both the FFR and FFI.

**Figure 1:**
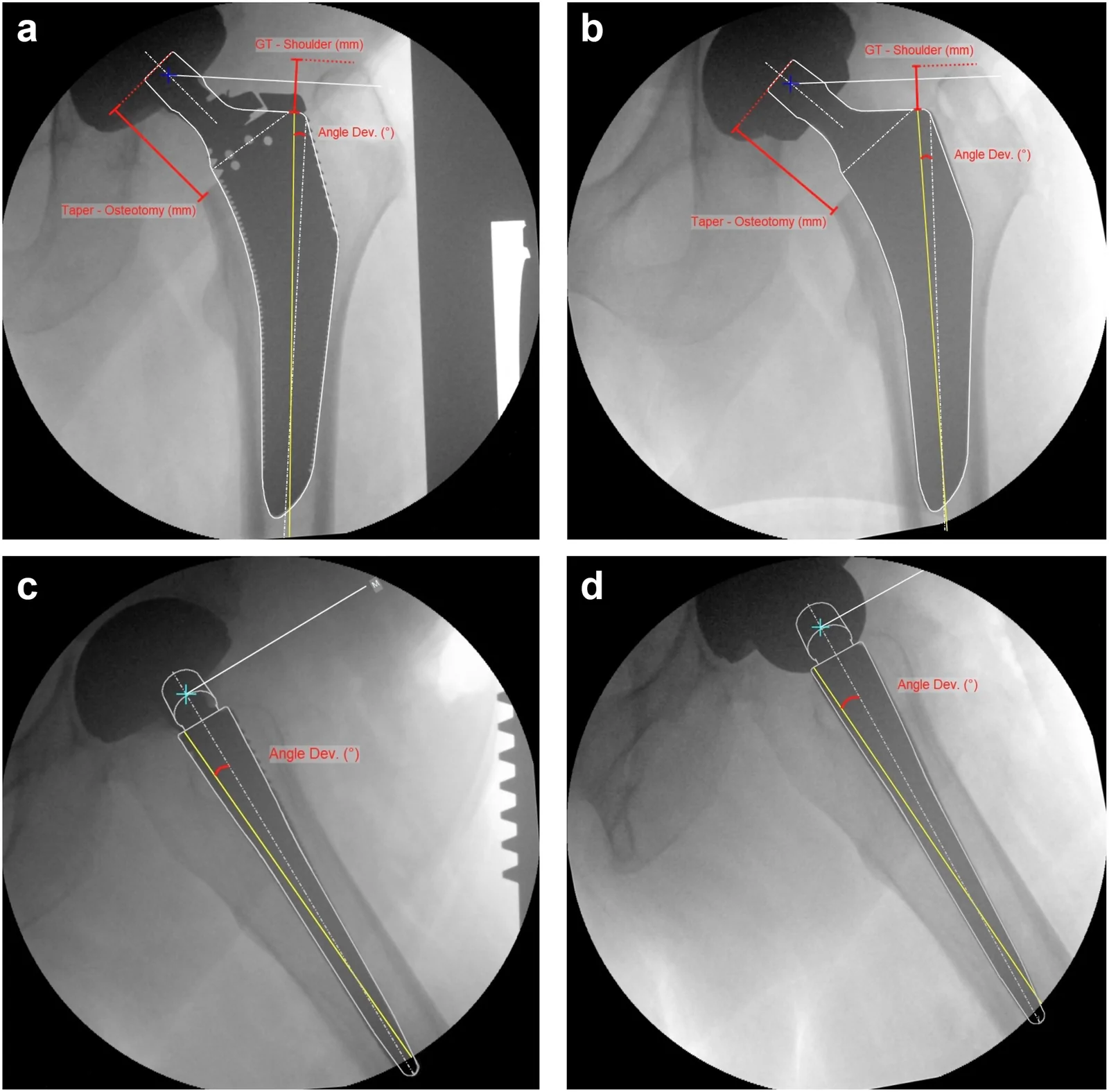
Intraoperative fluoroscopic assessment of the rasp and implant positions. (a) Rasp, coronal view (b) Implant, coronal view (c) Rasp, axial view (d) Implant, axial view. Measurements shown include GT–shoulder distance (mm), osteotomy–taper apex distance (mm) and angular deviation (°). The white dotted line indicates the longitudinal axis of the rasp/implant, whereas the yellow line indicates the FSA. dev., deviation.

Coronal measurements:

- Vertical distance from the osteotomy plane to the apex of the taper
- Angular deviation between the longitudinal axis and the FSA [11]
- Distance from the lateral shoulder to the tip of the greater trochanter (GT) [7,12]

Axial (Lauenstein) measurements:

- Angular deviation between the longitudinal axis and the FSA [13]

The positional differences between FFR and FFI were defined as Δ = FFR – FFI. Therefore, a positive value for the GT–shoulder distance reflected a more distal position of the FFR; for the osteotomy–taper distance, a more proximal position; and for the coronal angle deviation, a more varus alignment relative to the FSA. Angles were defined as positive when oriented in varus and negative when oriented in valgus relative to the FSA.

### Data analyses

Statistical analyses were performed using SPSS Statistics, Version 31.0 (IBM Corp., Armonk, NY, USA).

The sample size was determined by a priori power analysis (α = 0.05, power = 0.95) based on pilot data (Cohen’s d = 0.46), requiring 63 procedures to detect systematic coronal angle deviation between rasp and implant.

Normality was assessed using the Shapiro–Wilk test. Normally distributed data are presented as mean ± standard deviation and analyzed using parametric tests, whereas non-normally distributed data are reported as median and interquartile range (25th-75th percentiles). Positional differences between the FFR and FFI were assessed using one-sample *t-test*, group comparisons with independent-sample *t-test*, and associations with demographic or procedural variables using multivariable linear regression.

For intrarater reliability, 1 observer repeated the measurements for a randomized subset of 15 procedures after 14 days. Inter-rater reliability was assessed by three observers using the same subset under identical conditions. The subset size was consistent with empirical practice in similar radiographic reliability analyses [14]. Intraclass correlation coefficients (ICCs; two-way mixed model, absolute agreement) with 95% confidence intervals (CIs) were calculated.

## Results

### Analyses of final rasp and implant positioning

The positional measurements of the FFR and FFI in the coronal and axial views are presented in Table 2.

**Table 2:** Positional analyses of rasp and implant.

Parameter	FFR		FFI	
	Mean ± SD (Range)	95% CI	Mean ± SD (Range)	95% CI
Osteotomy–taper apex (mm)	38.6 ± 4.2 (31.3-47.3)	37.5-39.7	38.8 ± 4.3 (28.3-47.7)	37.7-39.8
Coronal angle deviation (°)	0.0 ± 1.9 (−4.0 to 5.8)	−0.5 to 0.4	−0.7 ± 1.8 (−4.3 to 4.6)	−1.1 to −0.2
GT–shoulder distance (mm)	9.8 ± 3.9 (1.0-17.6)	8.8-10.8	9.1 ± 3.8 (0.4-17.2)	8.2-10.1
Axial angle deviation (°)	4.8 ± 2.5 (0.0-11.3)	4.2-5.4	4.8 ± 2.6 (−0.1 to 11.8)	4.1-5.4

Values represent mean ± SD with ranges and corresponding 95% CIs for each parameter.

SD, standard deviation.

Mean angular and linear deviations indicated close agreement between the rasp and implant positioning.

The mean coronal angle deviation was 0.6° ± 1.4°, with the FFI positioned systematically more in valgus relative to the FSA compared with the FFR (*P* < .001; 95% CI 0.3-1.0°). The mean distance between the lateral shoulder and the tip of the GT was 0.6 ± 2.5 mm, showing a more proximal position of the FFI relative to the FFR (*P* = .048; 95% CI: 0.0-1.3 mm). The mean distance between the osteotomy plane and taper apex was −0.1 ± 1.7 mm, demonstrating no systematic deviation (*P* = .559; 95% CI, −0.6 to 0.3 mm). The mean axial angle deviation was 0.04° ± 1.59°, likewise without a significant difference (*P* = .836; 95% CI −0.36 to 0.44°).

The data distributions are illustrated in Figure 2.

**Figure 2:**
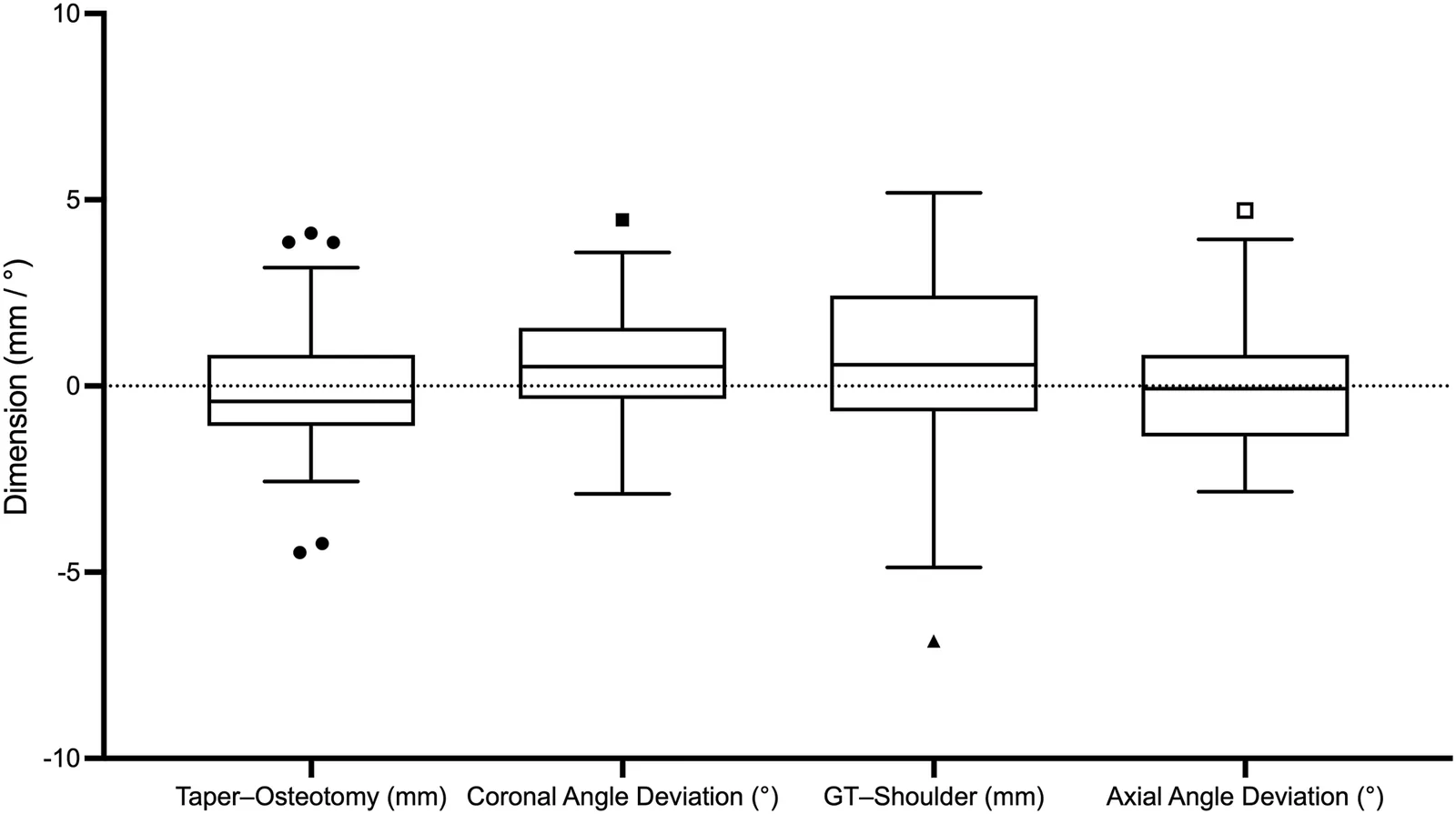
Differences between the rasp and implant positioning in the coronal and axial views. Boxplots showing the differences between the rasp and implant positioning for each measured parameter (Δ = FFR − FFI). Boxes represent the interquartile range, whiskers indicate 1.5 × IQR, and horizontal lines denote the median. The dashed line at zero represents an identical rasp–implant alignment. IQR, interquartile range.

### Determinants of rasp–implant positional differences

To identify factors associated with rasp–implant positional differences (Δ), multivariable linear regression was performed including patient-related variables (age, American Society of Anesthesiologists classification, sex, height, weight, body mass index, cortical thickness index [CTI]) and procedural variables (stem neck variant, stem size, surgeon experience). No significant associations were detected between these factors and rasp–implant mismatch.

Given its clinical relevance, surgeon experience was additionally analyzed descriptively. Coronal angle deviation did not differ significantly between procedures performed by highly experienced and experienced surgeons (0.5° ± 1.2° vs 0.8° ± 1.6°, *P* = .48), indicating comparable concordance across experience levels (Fig. 3).

**Figure 3:**
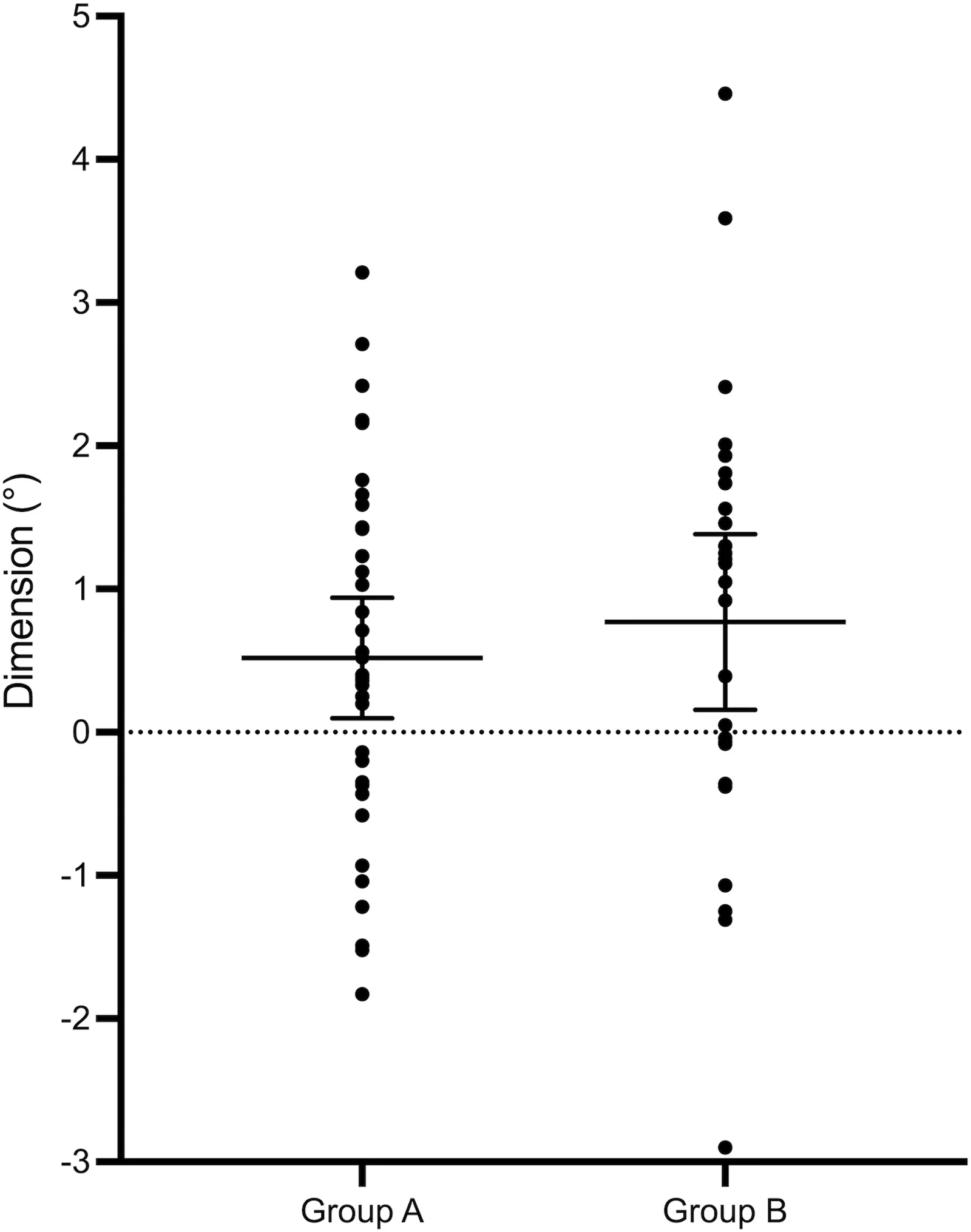
Coronal angle deviation difference between highly experienced and experienced surgeons. Scatter plot illustrating the coronal angle deviation difference (Δ = FFR − FFI, °) relative to the femoral shaft axis. Positive values indicate a more varus orientation of the FFR compared with the FFI; the dotted line at 0° represents perfect alignment concordance. Dots represent individual procedures, and horizontal lines denote group means with 95% CIs. Group A corresponds to highly experienced surgeons, and group B to experienced surgeons.

Likewise, comparison between standard and varus stem neck variants revealed no significant difference in coronal angle deviation (0.5° ± 1.5° vs 0.9° ± 1.0°, *P* = .25).

When analyzing component alignment independently, increasing stem size was significantly associated with a more varus orientation of the FFR (B = 0.60, 95% CI 0.35-0.85, *P* < .001) and the FFI (B = 0.48, 95% CI 0.23-0.73, *P* < .001). Likewise, a higher CTI was associated with more varus alignment of the FFR (B = 8.29, 95% CI 0.15-16.43, *P* = .046) and the FFI (B = 8.80, 95% CI 0.65-16.95, *P* = .035) (Fig. 4). These associations were observed for the alignment of the FFR and FFI individually but did not translate into increased rasp–implant mismatch.

**Figure 4:**
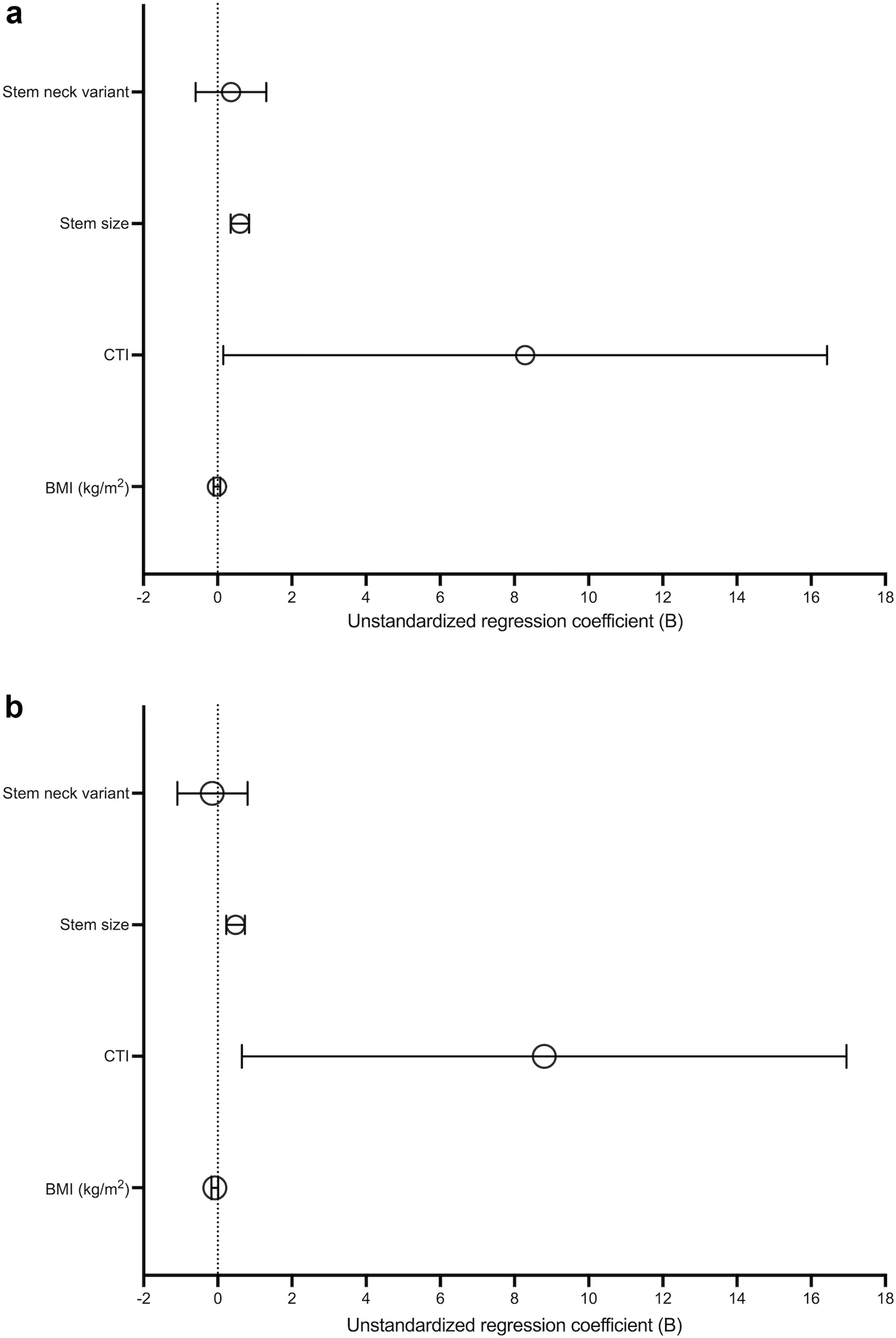
Multivariable linear regression models predicting coronal alignment of the final femoral rasp and final femoral implant. Forest plots illustrating unstandardized regression coefficients (b) with corresponding 95% CIs for the final femoral rasp (a) and final femoral implant (b). The vertical reference line at 0 indicates no effect. The reference category for the stem neck variant was the standard (132°) configuration. Model fit was R^2^ = 0.31 (adjusted R^2^ = 0.26, F(4, 58) = 6.43, *P* < .001) for the FFR model and R^2^ = 0.226 (adjusted R^2^ = 0.173, F(4, 58) = 4.23, *P* = .004) for the FFI model.

### Reliability analyses

An overview of the reliability analysis results is provided in Table 3. Intra-rater reliability demonstrated good agreement for the CTI and axial angle deviation (ICC = 0.89 and 0.77, respectively). ICC values for the osteotomy–taper apex distance, coronal angle deviation, and GT–shoulder distance ranged from 0.97 to 0.98, indicating excellent consistency.

**Table 3:** Overall ICC values of the outcome measures.

Parameter	Intra-rater reliability		Inter-rater reliability	
	ICC	95% CI	ICC	95% CI
CTI	0.89	0.71-0.96	0.93	0.84-0.97
Osteotomy–taper apex (mm)	0.98	0.96-0.99	0.95	0.92-0.98
Coronal angle deviation (°)	0.97	0.93-0.98	0.91	0.84-0.95
GT–shoulder distance (mm)	0.98	0.95-0.99	0.90	0.79-0.95
Axial angle deviation (°)	0.77	0.57-0.88	0.67	0.50-0.81

ICC calculated using a two-way mixed-effects model with absolute agreement (single measurement), reported with 95% CIs.

Inter-rater reliability among the three observers likewise showed ICC values ranging from 0.67 for axial angle deviation (moderate) to 0.95 detected for the osteotomy–taper apex distance (excellent) [15].

## Discussion

This study assessed intraoperative FFR and FFI positioning in cementless THA via the DAA using a triple-tapered stem. The results demonstrated close positional agreement between the FFR and the FFI, indicating that, within the present cohort, the intraoperative final rasp position reliably mirrored the definitive stem position. Systematic differences were identified in the coronal plane, with the FFI positioned on average more in valgus relative to the FSA and 0.6 mm more proximally than the trial rasp.

In terms of alignment, the definitive implant was positioned on average 0.6° more in valgus relative to the FSA than the final rasp, while the rasp itself was oriented close to neutral. This relative alignment difference was consistent across the cohort and independent of patient-related factors, femoral morphology, implant and procedural characteristics, suggesting that it reflected a systematic aspect of femoral preparation and implantation rather than variability explained by the assessed covariates. While a tendency toward varus positioning of straight or shoulder-type stems implanted via the DAA has previously been described [16], the reported data demonstrated that the definitive stem was, on average, oriented in slight valgus relative to the FSA. As THA generally strives for neutral coronal alignment of the femoral component relative to the anatomical axis [17] to promote even load distribution [18], this finding is unlikely to be intentional but may reflect subtle intraoperative adjustment during final implantation. Given this, intraoperative fluoroscopic assessment appears particularly relevant, enabling identification of alignment tendencies during femoral preparation and allowing targeted adjustment during definitive implantation, thereby mitigating the risk of stem malalignment [19]. Nevertheless, recent data indicate that slight valgus alignment of the definitive stem does not compromise clinical performance in cementless THA, suggesting that the minor deviations observed in the present study are unlikely to be clinically meaningful [17,20].

Moreover, a mean difference of 0.6 mm in the distance between the lateral shoulder and the GT was observed, with the definitive implant positioned more proximally than the final rasp, indicating a systematic height discrepancy between rasp and implant. This observation is consistent with previous reports describing predictable rasp–implant height discrepancies in cementless femoral stems [7]. This finding may be attributable to the presence of a titanium plasma coating of approximately 0.35 mm on the implant surface, which introduces a consistent geometric discrepancy compared with the rasp. In this context, Hofstaedter, Najfeld et al [7] reported a mean discrepancy of 2.6 mm in 39 patients, while Barink, Meijers et al [8] demonstrated mismatches below 2 mm in an experimental setup, neither of which were considered clinically relevant. Compared to these findings, the substantially smaller deviation observed in the present study is unlikely to translate into significant consequences. However, definitive cutoff values for rasp–implant mismatch remain undefined. Future studies should therefore investigate the relationship between mismatch extent and clinical outcomes.

An additional secondary finding was the association between increasing stem size and a more varus alignment of both the rasp and the definitive implant, emphasizing that stem alignment is largely determined during broaching rather than during the implantation step itself. Larger stem sizes may reflect anatomical constraints such as limited lateral bone stock or increased metaphyseal fill and may therefore promote varus orientation during femoral preparation. Although the CTI demonstrated statistical significance in the regression models, uncertainty regarding the magnitude of this effect limits its interpretability. Moreover, since relevant anatomical parameters known to influence femoral stem position, such as the critical trochanteric angle and the centrum–collum–diaphyseal angle, were not assessed, these results should be interpreted accordingly [16,21,22].

Taken together, these findings emphasize that fluoroscopic assessment of the final rasp provides a reliable basis for intraoperative decision-making. Potential deviations in alignment or implantation depth can be recognized and corrected before definitive stem implantation, whereas satisfactory rasp positioning provides confidence that the definitive implant will closely reproduce the planned position.

Several limitations should be acknowledged. The relatively large number of excluded procedures represents a potential source of selection bias. However, exclusions were exclusively related to incomplete fluoroscopic documentation or insufficient image quality rather than clinical characteristics, reflecting the predefined imaging quality criteria required for reliable paired radiographic measurements. Although a standardized surgical workflow was applied, minor intraoperative adjustments to optimize stem alignment may have occurred but were not systematically documented. This was particularly the case when the femoral rasp suggested a potential varus tendency, and minor additional reaming of the lateral cancellous bone was performed to optimize implant positioning. Furthermore, this study was conducted at a single high-volume institution using 1 contemporary triple-tapered stem design implanted exclusively through the DAA. Therefore, the present findings primarily apply to this specific implant system and surgical technique, and extrapolation to other stem geometries, fixation concepts, or surgical approaches should be made with caution. Finally, the analyses were based on two-dimensional intraoperative fluoroscopic images, which may not fully capture three-dimensional positional changes. Although strict fluoroscopic quality criteria were applied, minor rotational differences between sequential intraoperative image acquisitions cannot be completely excluded. Consequently, small projection-related measurement errors remain an inherent limitation of two-dimensional fluoroscopic analyses and should be considered when interpreting subtle angular differences.

However, the present study complements previous work by extending intraoperative rasp–implant analyses to multiple planes and provides the first multidimensional intraoperative quantification of rasp–implant mismatch in cementless THA. Given the clinical relevance of contemporary wedge-taper stem designs in cementless primary THA using the DAA, further studies are warranted to investigate the predictability of final implant positioning across different implant designs [9,23].

## Conclusions

High positional concordance between the FFR and the FFI was demonstrated. Systematic differences were observed, with the definitive stem positioned on average 0.6° more in valgus and 0.6 mm more proximally than the final rasp. These deviations most likely resulted from intraoperative adjustments to avoid malalignment as well as minor geometric differences between the rasp and implant design. Increasing stem size was associated with greater varus orientation of both the rasp and the definitive implant. Overall, these findings support intraoperative fluoroscopic assessment, particularly of the final rasp, to reduce the risk of implant malalignment and indicate that the final rasp provides a reliable intraoperative reference for stem positioning in cementless, triple-tapered stem THA performed via the DAA.

## Data availability

Data and materials are available from the corresponding author on reasonable request.

## Informed consent

The study was approved by the local ethics committee (Ref. 24-0188). As only anonymized routine clinical data were analyzed without study-specific interventions, individual informed consent was not required according to the ethics committee.

## Declaration of generative AI and AI-assisted technologies in the writing process

During the preparation of this work, the authors used ChatGPT (OpenAI) to improve language clarity and readability. After using this tool, the authors reviewed and edited the content as needed and take full responsibility for the content of this article.

## CRediT authorship contribution statement

**Yannick Alexander Ahlheit:** Writing – review & editing, Writing – original draft, Visualization, Validation, Supervision, Methodology, Investigation, Formal analysis, Data curation, Conceptualization. **Gautier Beckers:** Writing – review & editing, Validation, Supervision, Project administration, Investigation, Conceptualization. **Dominic Simon:** Validation, Supervision, Investigation. **Salome Hagelstein:** Methodology, Investigation. **Mattia De Benedictis:** Methodology, Investigation. **Boris Michael Holzapfel:** Writing – review & editing, Validation, Supervision, Resources, Investigation, Conceptualization. **Lennart Marvin Schroeder:** Writing – review & editing, Validation, Supervision, Project administration, Methodology, Investigation, Conceptualization.

## Conflicts of interest

Boris Michael Holzapfel receives honoraria for speakers bureau activities or paid presentations for B.Braun/Aesculap, ENOVIS, Bonesupport, Symbios; is a paid consultant for B.Braun/Aesculap, ENOVIS, Bonesupport, Symbios; Research support from a company or supplier as a Principal Investigator for Implantcast, Medacta, ZimmerBiomet, Bonesupport; serves as a board or committee member for DGOU, DGOOC, DCGOU, DGH, AE. All other authors declare no potential conflicts of interest.

For full disclosure statements refer to https://doi.org/10.1016/j.artd.2026.102186.

## Appendix A Supplementary data

Conflict of Interest Statement for Holzapfel

Conflict of Interest Statement for Simon

Conflict of Interest Statement for Beckers

Conflict of Interest Statement for Schroeder

Conflict of Interest Statement for De Benedictis

Conflict of Interest Statement for Hagelstein

Conflict of Interest Statement for Ahlheit
