# Supplementary material for: Final Femoral Rasp Predicts Intraoperative Stem Alignment in Primary Total Hip Arthroplasty Using a Cementless Triple-Tapered Stem
Source: Arthroplast Today. 2026 Oct 15;42:102186. doi: 10.1016/j.artd.2026.102186 (PMC13694775; doi:10.1016/j.artd.2026.102186)
Supplement: Supplementary file 1 [file mmc1.pdf]

INDIVIDUAL CONFLICT OF INTEREST STATEMENT

American Association of Hip and Knee Surgeons

(Adopted from the American Academy of Orthopaedic Surgeons disclosure statement)

The following form **must be filled out completely and submitted by each author (example, 6 authors, 6 forms). All items require a response. If there is no relevant disclosure for a given item, enter "*None*."**

Manuscript Title:

Final Femoral Rasp Predicts Definitive Stem Alignment in Cementless Triple-Tapered Short-Stem Total Hip Arthroplasty

---

1. Royalties from a company or supplier (The following conflicts were disclosed)
None

2. Speakers bureau/paid presentations for a company or supplier (The following conflicts were disclosed)
Paid presentations for B.Braun/Aesculap, ENOVIS, Bonesupport,Symbios

3A. Paid employee for a company or supplier (The following conflicts were disclosed)
None

3B. Paid consultant for a company or supplier (The following conflicts were disclosed)
Consultant for B.Braun/Aesculap, ENOVIS, Bonesupport, Symbios

3C. Unpaid consultants for a company or supplier (The following conflicts were disclosed)
None

4. Stock or stock options in a company or supplier (The following conflicts were disclosed)
None

5. Research support from a company or supplier as a Principal Investigator (The following conflicts were disclosed)
Institutional research support from Implantcast, Medacta, ZimmerBiomet, Bonesupport

6. Other financial or material support from a company or supplier (The following conflicts were disclosed)
None

7. Royalties, financial or material support from publishers (The following conflicts were disclosed)
None

8. Medical/Orthopaedic publications editorial/governing board (The following conflicts were disclosed)
None

9. Board member/committee appointments for a society (The following conflicts were disclosed)
DGOU, DGOOC, DCGOU, DGH, AE

Each author must sign AND print or type his/her name, date and submit a separate form

In addition, one BLINDED Conflict of Interest form (no author names used) should be submitted per manuscript with all author disclosures.

Boris Michael Holzapfel	[signature]	30.03.2026
Author Name (Print or Type)	Author Signature	Date
